# Analysis of Risk Factors for White Matter Hyperintensity in Older Adults without Stroke

**DOI:** 10.3390/brainsci13050835

**Published:** 2023-05-22

**Authors:** Kai Zheng, Zheng Wang, Xi Chen, Jiajie Chen, Yu Fu, Qin Chen

**Affiliations:** Department of Geriatrics, Tongji Hospital, Tongji Medical College, Huazhong University of Science and Technology, 1095 Jiefang Avenue, Wuhan 430030, China

**Keywords:** age, hypertension, homocysteine, proteinuria, white matter hyperintensity, nonstroke patient, magnetic resonance imaging

## Abstract

Background: White matter hyperintensity (WMH) is prevalent in older adults aged 60 and above. A large proportion of people with WMH have not experienced stroke and little has been reported in the literature. Methods: The case data of patients aged ≥60 years without stroke in Wuhan Tongji Hospital from January 2015 to December 2019 were retrospectively analyzed. It was a cross-sectional study. Univariate analysis and logistic regression were used to analyze independent risk factors for WMH. The severity of WMH was assessed using the Fazekas scores. The participants with WMH were divided into periventricular white matter hyperintensity (PWMH) group and deep white matter hyperintensity (DWMH) group, then the risk factors of WMH severity were explored separately. Results: Eventually, 655 patients were included; among the patients, 574 (87.6%) were diagnosed with WMH. Binary logistic regression showed that age and hypertension were associated with the prevalence of WMH. Ordinal logistic regression showed that age, homocysteine, and proteinuria were associated with the severity of WMH. Age and proteinuria were associated with the severity of PWMH. Age and proteinuria were associated with the severity of DWMH. Conclusions: The present study showed that in patients aged ≥60 years without stroke, age and hypertension were independent risk factors for the prevalence of WMH; while the increasing of age, homocysteine, and proteinuria were associated with greater WMH burden.

## 1. Introduction

White matter hyperintensity (WMH) refers to the hyperintensity on the T2 or fluid-attenuated inversion recovery (FLAIR) sequence, and the isointense or hypointense in T1 sequence, which is mostly distributed in the periventricular area, deep white matter, basal ganglia (deep gray matter), pons, and occasionally in the brain stem and other parts of the white matter of the cerebellum [1,2]. It has been shown that the overall prevalence of WMH in young people is 25.94% and increases with age, with 85.49% of the WMH being mild and located mainly in the frontal lobe and parietal subcortical white matter [3]. The prevalence of WMH in older adults is higher compared with the younger population. The Rotterdam Scan Study reported that only 5% of participants aged 60–90 years had no WMH at all, and the prevalence was highly correlated with age [4]. A study based on Chinese population demonstrated that the prevalence of periventricular white matter hyperintensity (PWMH) was 72.1%, including 87.7% in the 60–69 age group and 97.1% in the 70–80 age group; the prevalence of deep white matter hyperintensity (DWMH) was 65.4%, including 83.2% in the 60–69 age group and 89.5% in the 70–80 age group.

WMH is not associated with specific symptoms, but it does have an inextricable relationship with stroke, cognitive decline, dementia, motor impairment, depression, and vascular Parkinsonism [5,6]. It was one of the most common imaging markers in cerebral small vessel disease. The two most common pathologies of cerebral small vessel disease are arteriolosclerosis caused by aging, hypertension, and other conventional vascular risk factors, and cerebral amyloid angiopathy (CAA) [7]. Since the treatment of cerebral small vessel disease is limited according to the limited understanding of pathophysiology, we need to explore more risk factors, etiology, and mechanisms to treat and prevent this disease.

WMH is an independent risk factor for stroke, and the larger the volume of WMH, the worse the prognosis of stroke [8,9]. A recent meta-analysis indicated that severe WMH was associated with a significantly increased risk of cognitive impairment and all-cause dementia [10]. In addition to the severity of WMH, the location of WMH is also associated with cognitive impairment, dementia, and stroke [11,12,13,14,15]. Patients with PWMH were reported to have a significantly higher rate of cognitive decline than normal and mild patients, whereas there was no significant association between the severity of DWMH and cognitive function [16,17]. Similarly, PWMH was associated with an increased risk of all-cause dementia, and there was no significant association between DWMH and dementia risk; PWMH was associated with functional stroke outcomes, but DWMH was not [18,19]. Previous studies on WMH risk factors tended to focus on groups with a history of, or currently experiencing, stroke, but parts of WMH without a history of stroke will progress to stroke or dementia, and the rate of progression of early WMH was significantly lower than that of late [20], so the identification and prevention of WMH in older adults without stroke is particularly important. This study investigated the risk factors of WMH in patients of older adults aged 60 and above without stroke history based on the data of inpatients in a large tertiary hospital. This hospital-based cross-sectional study answered three main questions: independent risk factors for WMH, independent risk factors for the severity of WMH, and independent risk factors for the severity of WMH at different sites.

## 2. Methods

### 2.1. Study Population

This survey is a retrospective study based on the inpatient population conducted at Tongji Hospital in Wuhan, China. We included a total of 655 eligible patients who were hospitalized in the Department of Neurology of Tongji Hospital from January 2015 to December 2019, including 574 in the WMH group and 81 in the control group (without WMH). Inclusion criteria: (1) Age ≥ 60 years old. (2) Magnetic resonance images (MRI) records are available. Exclusion criteria: (1) Have moderate to severe cognitive impairment or dementia (DSM-IV-R). (2) History or current other cerebrovascular diseases (transient ischemic attack, stroke, intracranial hemorrhage, etc.). (3) Patients with WMH caused by factors other than age and vascular factors (such as patients who were diagnosed with multiple sclerosis, CADASIL, CARASIL, Fabry disease or MELAS syndrome); patients with encephalitis, epilepsy, traumatic brain injury, cancer, or severe psychiatric illness were also excluded. (4) Have serious heart, lung, liver, kidney disease, tumors, etc. (5) Incomplete data. This study complies with the Declaration of Helsinki. The study protocol was reviewed and approved by the Ethics Committee of Tongji Hospital, Tongji Medical College, Huazhong University of Science and Technology, and was exempted from the requirement of informed consent because it is a retrospective study. The clinical trial registration number is ChiCTR2100049617 (https://www.chictr.org.cn/, accessed on 6 June 2021).

### 2.2. Data Collection and Evaluation

The basic information and clinical data of the research subjects were collected and calculated through the electronic medical record system of Tongji Hospital. Variables collected included age, gender, hypertension, diabetes, smoking, hypersensitive C-reactive protein (hs-CRP), total cholesterol (TC), high-density lipoprotein (HDL), low-density lipoprotein (LDL), triglycerides, alkaline phosphatase, uric acid, cystatin C, homocysteine, high-sensitivity troponin I (hsTnI), proteinuria, and Framingham score (FHS). The FHS adopted the version published in 2008 to assess the risk of cardiovascular disease in subjects over a 10-year period [21], which included items such as age, sex, HDL, smoking, diabetes, and systolic blood pressure. Hypertension included a previous history of hypertension or hospital measurement of systolic blood pressure (SBP) ≥ 140 mmHg and/or diastolic blood pressure (DBP) ≥ 90 mmHg [22]. Diabetes mellitus was defined as previously diagnosed or fasting glucose ≥ 7.0 mmol/L and/or random blood glucose ≥ 11.1 mmol/L [23]. The classification of smoking was based on patient self-report, and smoking was defined as continuous or cumulative smoking for more than 6 months in a lifetime. Proteinuria was defined as ≥150 mg of protein in excreted urine per day [24].

### 2.3. Acquisition and Analysis of MRI

**Figure 1:**
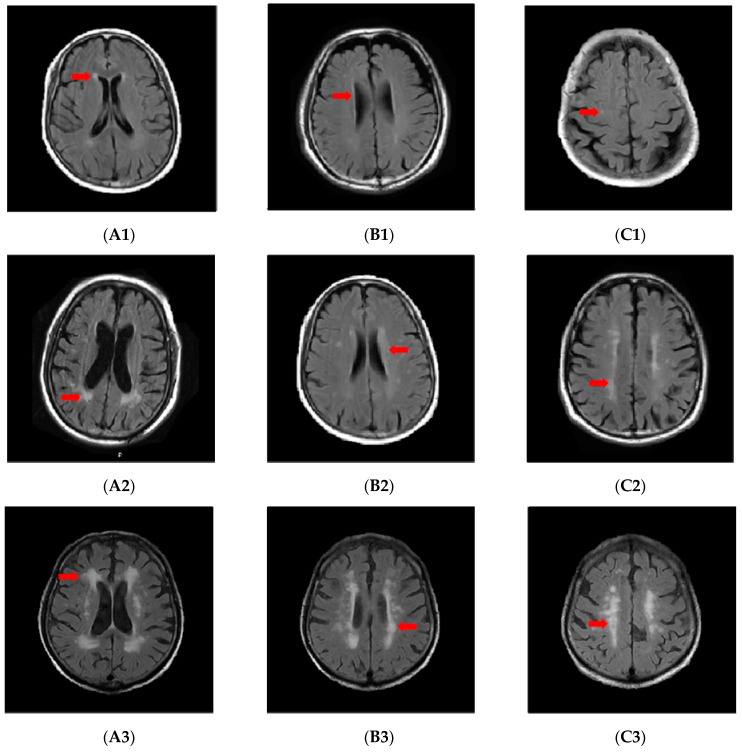
PWMH with Fazekas score of 1 (**A1**,**B1**); DWMH with Fazekas score of 1 (**C1**); PWMH with Fazekas score of 2 (**A2**,**B2**); DWMH with Fazekas score of 2 (**C2**); PWMH with Fazekas score of 3 (**A3**,**B3**); DWMH with Fazekas score of 3 (**C3**). The area indicated by the arrow in the figure is typical white matter hyperintensity. PWMH, periventricular white matter hyperintensity; DWMH, deep white matter hyperintensity.

All patients’ MRI images were acquired in the Tongji hospital using a 1.5 Tesla MRI scanner. WMH is defined as the abnormal signal in the white matter region in MRI images, which manifested by high signal on T2 and FLAIR sequences and isointense or hypointense on T1 sequences. Fazekas score was used to classify the severity of WMH, and the scoring criteria are shown below (Figure 1). Periventricular white matter: 0 = none; 1 = cap or pencil-like thin lesion; 2 = smooth “halo”; 3 = irregular periventricular signal extending into deep white matter. Deep white matter: 0 = absent; 1 = single or multiple spots; 2 = beginning confluence; 3 = large areas of confluence. We defined a score of 0 as no WMH, 1 as mild, 2 as moderate, and 3 as severe. The more severe grade of periventricular white matter and deep white matter was taken as the severity of the overall WMH. Two experienced neurologists independently evaluated the MRI images with blind to any other information about the patient, and when disagreements occurred, a third neurologist was asked to make the final diagnosis.

### 2.4. Statistical Analysis

All of the analytical procedures were completed in SPSS 25.0 with a two-sided test and statistical significance when *p* < 0.05. In the baseline data, categorical variables are represented by the number of events and percentages. If continuous variables are in a normal distribution, they are represented by the mean ± standard deviation, otherwise they are expressed as median (first quartile, third quartile). The included study subjects were divided into two groups according to the existence of WMH (case and control), and then univariate analysis was used to test the connection between WMH and potential related factors. The relationship of SBP, DBP, glucose, TC, HDL, LDL, alkaline phosphatase, cystatin C, uric acid, and FHS with WMH was analyzed by *t*-test. χ^2^ test was used to analysis the connection between WMH and gender, hypertension, diabetes, smoking, and proteinuria. Mann–Whitney U-test was used to examine the association between WMH and age, hs-CRP, triglycerides, homocysteine, and hsTnI. Binary logistic regression was used to identify independent risk factors for WMH, adjusting for hypertension, age, systolic blood pressure, diastolic blood pressure, antihypertensive medication use, smoking, diabetes mellitus, fasting glucose, glucose-lowering medication or insulin use, TC, cystatin C, proteinuria, homocysteine, and FHS. The patients with WMH were divided into three groups according to the severity, Patients in the WMH group were then divided into three groups according to severity, and one-way ANOVA or Kruskal–Wallis test was selected for analysis depending on the type and distribution of the data. On the basis of the results of the univariate analysis, statistically significant independent variables were included in an ordinal logistic regression, which resulted in independent factors influencing the severity of WMH. To compare the independent risk factors for WMH severity at different locations, we performed ordinal logistic regression for PWMH and DWMH separately. The relationship between risk factors and WMH was evaluated by odds ratios (ORs) with 95% confidence intervals (CIs) and *p* values.

## 3. Results

### 3.1. Risk Factors of Prevalence of WMH

A total of 655 patients were included according to the inclusion and exclusion criteria, and their demographic and clinical data are summarized in Table 1. Of the 655 study participants, the age range was 60–93 years, with a median of 67 years; 574 (87.6%) had WMH; 319 (48.7%) had hypertension, of which 271 (85.0%) were treated; 85 (13.0%) had diabetes, of which 71 (83.5%) were treated. The basic characteristics and univariate analysis results of the WMH group and the control group are shown in Table 2. The differences in age (*p* = 0.001), hypertension (*p* < 0.001), cystatin C (*p* = 0.039), and FHS (*p* = 0.015) were statistically significant between the two groups, which means that age, hypertension, cystatin C, and FHS can be considered as risk factors for WMH in this study. After adjusting for confounding factors such as age, hypertension, SBP, DBP, diabetes, fasting glucose, smoking, TC, cystatin C, homocysteine, proteinuria, and FHS, binary logistic regression showed that hypertension (OR = 2.827, 95% CI = 1.513~5.283, *p* = 0.001) and age (OR = 1.069, 95% CI = 1.011~1.129, *p* = 0.018) were significantly associated with WMH (Table 1). The results indicated that hypertension and age were independent risk factors for WMH.

### 3.2. Risk Factors for the Severity of WMH

Patients with WMH were divided into mild group (*n* = 338), moderate group (*n* = 177), and severe group (*n* = 59), according to the Fazekas score. Univariate analysis suggested that age, gender, smoking, hypertension, TC, LDL, cystatin C, hsTnI, homocysteine, and FHS are risk factors for the severity of WMH (Table 3). Because of the interaction between TC and LDL, we excluded TC and retained the more relevant LDL based on clinical experience. After adjusting for confounding factors such as age, sex, hypertension, smoking, LDL, cystatin C, hsTnI, proteinuria, homocysteine, and FHS, ordinal logistic regression showed that the differences in age (OR = 1.057, 95% CI = 1.038~1.076, *p* < 0.001), homocysteine (OR = 1.017, 95% CI = 1.002~1.033, *p* = 0.026), and proteinuria (OR = 0.628, 95% CI = 0.474~0.832, *p* = 0.001) among the three groups remained statistically significant (Table 2). The results indicated that age, homocysteine, and proteinuria were independent risk factors for the severity of WMH.

### 3.3. Risk Factors for the Severity of PWMH

Patients with PWMH were divided into mild group (*n* = 336), moderate group (*n* = 168), and severe group (*n* = 35), according to the Fazekas score. Univariate analysis suggested that age, male, smoking, hypertension, cystatin C, homocysteine, and FHS were risk factors for the severity of PWMH (Table 4). After adjusting for confounding factors such as age, hypertension, smoking, cystatin C, proteinuria, homocysteine, and FHS, ordinal logistic regression showed that the differences in age (OR = 1.079, 95% CI = 1.044~1.114, *p* < 0.001) and proteinuria (OR = 0.587, 95% CI = 0.359~0.670, *p* = 0.034) among the three groups remained statistically significant (Table 3). The results indicated that age and proteinuria were independent risk factors for the severity of PWMH.

### 3.4. Risk factors for the Severity of DWMH

Patients with DWMH were divided into mild group (*n* = 407), moderate group (*n* = 110), and severe group (*n* = 50), according to the Fazekas score. Univariate analysis suggested that age, smoking, hypertension, cystatin C, homocysteine, hsTnI, proteinuria, uric acid, and FHS were risk factors for the severity of DWMH (Table 5). After adjusting for confounding factors such as age, hypertension, smoking, cystatin C, homocysteine, hsTnI, proteinuria, uric acid, and FHS, ordinal logistic regression showed that the differences in age (OR = 1.090, 95% CI = 1.053~1.127, *p* < 0.001) and proteinuria (OR = 0.397, 95% CI = 0.239~0.658, *p* < 0.001) among the three groups remained statistically significant (Table 4). The results indicated that age and proteinuria were independent risk factors for the severity of DWMH.

## 4. Discussion

This is a cross-sectional study that concentrated on exploring the risk factors of the prevalence of WMH and severity of WMH in the non-stroke hospitalized patient (e.g., dizziness, headache, mild cognitive function, depression, sleep disorder, or Parkinsonism) in the middle area of China.

In our study we found that in older adults aged 60 and above without stroke, hypertension and age were independently associated with the prevalence of WMH. It was consistent with the previous studies of general population without stroke, but in all hospitalized patient without stroke, besides age and hypertension, diabetes mellitus and smoking and HCY levels were also shown as risk factors of occurrence of WMH [25,26,27,28,29]. This may be due to the different diagnosed inpatients that were adopted in the hospital, and that our patient number was limited. However, age and hypertension were always the most common risk factors.

In this study, we also found that age, homocysteine, and proteinuria were independently associated with the severity of WMH; age and proteinuria were independently associated with the severity of PWMH; and age and proteinuria were independently associated with the severity of DWMH in our study. It is a little different to severity risk factors (smoking, hypertension, and male sex) of some previous studies [26,28]. This result might have been caused by the small sample size and the cross-sectional nature of the study. Although the investigation design is not that complete, there is now more and more evidence showing that chronic kidney insufficiency manifested as low eGFR, proteinuria, albuminuria, and elevated serum cystatin C was independently associated with prevalence and severity of WMH, especially in DWMH [30,31]. Many studies showed that chronic kidney disease is strongly associated with cerebral small vessel disease, and related to clinical symptoms such as cognitive impairment, dementia, and stroke.

As we know, age is one of the most common risk factors for the prevalence and severity of WMH [25,26,27,28,29], and our study supports this view as well. In our study, we found that (1) the patients with WMH were older relative to those with normal brain MRI; (2) in the group with WMH, participants with high-score WMH were older than those with low-score WMH; (3) WMH was divided into PWMH and DWMH, and age was associated with the severity of DWMH and PWMH, respectively; and all of the above relationships were statistically significant. The associations between age and WMH may be influenced by the age distribution and health status of the study population. Kevin S. King et al. found that WMH volumes increased more rapidly with age in the comorbid group (presence of hypertension, diabetes, and obesity) at age ≥ 50 years, with a significantly greater difference in the expected WMH age slope compared with the normal group [32]. A study by Ana R. Moura demonstrated that 43 years was the inflection point for the relationship between age and WMH volume, which became closer to linear at age ≥ 43 years [33]. In conclusion, WMH volume is positively correlated with age, and when age reaches a certain inflection point, WMH volume increases rapidly with age. It is worth noting that age tends to be an independent risk factor for WMH, both in healthy populations and in various disease groups, and this association is unrelated to the location of WMH.

Hypertension was another independent risk factor for the prevalence of WMH, but not for the severity of WMH in this study. A study that included 10 European cohorts of 1625 nondemented subjects demonstrated that people with hypertension were at higher risk of severe WMH than those without hypertension [34]. It has been suggested that the association between hypertension and WMH depends on age, and in the predementia and mild dementia groups, total WMH was higher in people with hypertension compared to those without hypertension, but this association was only statistically significant between the age groups of 60–69 years [35]. In conclusion, hypertension is positively associated with WMH, but the strength of this association may be age-related. It is possible that cerebral vascular morphology mediates the relationship between blood pressure and WMH [36]. Hypertension causes a decrease in the density and number of small vessel distribution, leading to inadequate perfusion of small arteries and consequent small vessel disease [37,38].

Homocysteine can cause vascular endothelial dysfunction by increasing lipid peroxidation through the production of reactive oxygen species [39,40]. In addition, homocysteine decreases endothelial nitric oxide bioavailability and enhances vascular inflammation, thereby disrupting the integrity of the blood–brain barrier [41,42]. Homocysteine was shown to be an independent predictor of WMH volume, and patients with high-grade WMH had significantly higher homocysteine levels than those with low-grade WMH [43]. The present study also supports these views, while further analyzing the effect of WMH location on the association between homocysteine and WMH. Unexpectedly, homocysteine was independently associated with overall WMH severity in the stroke-free older adults, which was not influenced by the location of WMH.

We always assume that kidney and brain have similar microvascular structures, and the presence of proteinuria indicates increased permeability of the vascular membrane and impaired endothelial function [44,45], which may explain why patients with proteinuria are more likely to develop high-grade WMH, but, actually, the underlying pathophysiological mechanism is still poorly understood. In our current cross-sectional study, proteinuria was an independent risk factor for WMH severity, and further studies found that this association remained constant both in the DWMH group and in the PWMH group. In a retrospective community-based study, patients with albuminuria were found to be at higher risk of increased WMH relative to normal individuals [46]. Sang Hyuck Kim et al. found that prominent albuminuria was significantly associated with increased WMH volume [47]. Chronic kidney disease is often asymptomatic until it progresses to an advanced stage, but mild and moderate chronic kidney disease triggers various pathogenic mechanisms such as inflammation, oxidative stress, neurohormonal imbalance, formation of uremic toxins and vascular calcification, followed by damage to the endothelium and blood vessels [48].

## 5. Strength and Limitations

This was a pilot study in which we explored the risk factors of cerebral small vessel diseases in nonstroke hospitalized patients, and for the first time, we found that proteinuria may be strongly associated with the severity of WMH.

Although the study was analyzed in detail, there are still some limitations. In assessing the severity of WMH, the Fazekas visual scale can only be used as a substitute for WMH volumetric measurements due to limitations, which may be somewhat subjective. As the study was a cross-sectional study, it was not possible to determine the causal relationship between the factors and WMH. In addition, due to the limitation of objective conditions, this study did not describe the symptoms and detail diagnosis of the patients, but MRI, as an important detection method in the neurology department, was used in most of the hospitalized patients in the neurology department. Although none of the enrolled individuals had a history of stroke, this is the result of 655 patients at one hospital and is not representative of the general population. We must admit that when analyzing the risk factors for the severity of PWMH(DWMH), these patients had DWMH(PWMH) as well simultaneously; however, based on the data and clinical experience, we found that it is impossible to separate DWMH from PWMH, because WMH is a whole lesion of white matter, rather than a single location.

In summary, we suggest that future studies with large sample sizes should be carried out, and we could investigate more detailed indicators of kidney function and design more convincing longitudinal evidence for the association between WMH and kidney function to deeply investigate the associations of kidney disease and WMH.

## 6. Conclusions

This was a pilot study which enrolled hospitalized patients aged over 60 without stroke. We found that age and hypertension were independent risk factors for the prevalence of WMH; age and proteinuria were independent risk factors for the severity of PWMH and DWMH. Proteinuria provided a relatively new insight to highlight the association of chronic kidney disease and cerebral small vessel disease in the elderly population and to explore more mechanisms in the future. We could find many subclinical cerebral abnormalities in the chronic kidney disease patient before they have neurology symptoms. We may continue to proceed with more rigorous design investigation and large-sample, high-quality longitudinal studies, to provide more persuasive evidence. While treating risk factors such as hypertension will reduce WMH risk, more risk factors and targets for treatment and prevention will be found in the future. It would be exciting work to reduce the enormous cerebral small vessel disease and later impact as early as possible.

## Figures and Tables

**Table 1:** Characteristics of the study population.

Characteristic	Total Participants (*n* = 655)
Age, y	67 (62, 72)
Male, *n* (%)	315 (48.1)
WMH, *n* (%)	574 (87.6)
Hypertension, *n* (%)	319 (48.7)
SBP, mmHg	134.7 ± 18.8
DBP, mmHg	79.9 ± 10.9
Diabetes, *n* (%)	85 (13.0)
Fasting glucose, mmol/L	5.48 ± 1.39
Smoking history, *n* (%)	136 (20.8)
hs-CRP, mg/L	0.9 (0.5, 2.3)
Total cholesterol, mmol/L	4.07 ± 0.98
LDL, mmol/L	2.50 ± 0.81
HDL, mmol/L	1.13 ± 0.29
Triglycerides, mmol/L	1.09 (0.82, 1.53)
Alkaline phosphatase, U/L	68.0 ± 21.1
Cystatin C, mg/L	1.08 ± 0.27
Homocysteine, μmol/L	12.3 (9.9, 12.5)
Uric acid, μmol/L	302.50 ± 86.83
HsTnI, pg/mL	3.7 (2.2, 7.6)
Proteinuria, *n* (%)	87 (13.3)
FHS	15.49 ± 4.50

Note. Data presented as mean ± SD or median (Q1, Q3) for continuous variables, and *n* (%) for categorical variables. WMH, white matter hypertension; SBP, systolic blood pressure; DBP, diastolic blood pressure; hs-CRP, hypersensitive C-reactive protein; LDL, low-density lipoprotein; HDL, high-density lipoprotein; HsTnI, high-sensitivity troponin I; FHS, Framingham score.

**Table 2:** Univariate analysis of risk factors of WMH.

Characteristic	Control (*n* = 81)	WMH (*n* = 574)	*p*
Age, year	64.0 (61.0, 68.5)	67 (63, 72)	0.001
Male, *n* (%)	36 (44.4)	279 (48.6)	0.483
Hypertension, *n* (%)	23 (28.4)	296 (51.6)	<0.001
SBP, mmHg	132.95 ± 16.24	134.97 ± 19.12	0.365
DBP, mmHg	79.37 ± 10.63	79.99 ± 10.91	0.633
Diabetes, *n* (%)	7 (8.6)	78 (13.6)	0.215
FBG, mmol/L	5.52 ± 1.32	5.47 ± 1.40	0.748
Smoking, *n* (%)	16 (19.8)	120 (20.9)	0.811
hs-CRP, mg/L	0.90 (0.50, 2.55)	0.90 (0.50, 2.20)	0.957
TC, mmol/L	4.24 ± 0.93	4.05 ± 0.99	0.106
LDL, mmol/L	2.58 ± 0.84	2.49 ± 0.81	0.347
HDL, mmol/L [11]	1.15 ± 0.33	1.12 ± 0.29	0.512
Triglycerides, mmol/L	1.16 (0.89, 1.49)	1.08 (0.80, 1.54)	0.385
Alkaline phosphatase, U/L	67.91 ± 17.52	68.03 ± 21.60	0.961
Cystatin C, mg/L	1.02 ± 0.23	1.09 ± 0.27	0.039
Homocysteine, μmol/L	11.80 (9.20, 13.85)	12.40 (9.90, 15.40)	0.074
Uric acid, μmol/L	309.92 ± 94.16	301.41 ± 85.78	0.409
HsTnI, pg/mL	2.80 (1.90, 6.10)	3.75 (2.20, 7.60)	0.085
Proteinuria, *n* (%)	8 (9.9)	79 (13.8)	0.335
FHS	14.36 ± 4.15	15.65 ± 0.19	0.015

Note. Data presented as mean ± SD or median (Q1, Q3) for continuous variables, and *n* (%) for categorical variables. WMH, white matter hypertension; SBP, systolic blood pressure; DBP, diastolic blood pressure; FBG, fasting blood glucose; hs-CRP, hypersensitive C-reactive protein; TC, total cholesterol; LDL, low-density lipoprotein; HDL, high-density lipoprotein; HsTnI, high-sensitivity troponin I; FHS, Framingham score.

**Table 3:** Univariate analysis of risk factors for the severity of WMH.

Characteristic	Mild WMH (*n* = 338)	Moderate WMH (*n* = 177)	Severe WMH (*n* = 59)	*p*
Age, year	65.0 (62.0, 70.0)	69.0 (65.0, 73.5)	72.0 (67.0, 80.0)	<0.001
Male, *n* (%)	160 (47.3)	80 (45.2)	39 (66.1)	0.016
Smoking, *n* (%)	63 (17.8)	37 (20.9)	20 (33.9)	0.029
Hypertension, *n* (%)	157 (46.4)	100 (56.5)	39 (64.4)	0.006
TC, mmol/L	4.13 ± 1.01	3.85 ± 0.90	4.17 ± 1.05	0.005
LDL, mmol/L	2.54 ± 0.82	2.35 ± 0.77	2.57 ± 0.83	0.032
Cystatin C, mg/L	1.01 (0.88, 1.19)	1.05 (0.92, 1.25)	1.13 (0.96, 1.34)	0.001
HsTnI, pg/mL	3.60 (2.10, 7.40)	3.90 (2.30, 7.50)	4.60 (3.10, 10.00)	0.005
Proteinuria, *n* (%)	34 (10.1)	33 (18.6)	12 (20.3)	0.008
HCY, μmol/L	11.95 (9.60, 14.80)	12.80 (10.25, 15.65)	15.20 (11.40, 20.20)	<0.001
FHS	14.98 ± 4.50	16.11 ± 4.32	18.14 ± 4.38	<0.001

Note. Data presented as mean ± SD or median (Q1, Q3) for continuous variables, and *n* (%) for categorical variables. WMH, white matter hypertension; TC, total cholesterol; LDL, low-density lipoprotein; HsTnI, high-sensitivity troponin I; HCY, homocysteine; FHS, Framingham score.

**Table 4:** Univariate analysis of risk factors for the severity of PWMH.

Characteristic	Mild PWMH (*n* = 336)	Moderate PWMH (*n* = 168)	Severe PWMH (*n* = 35)	*p*
Age, year	66.0 (62.0, 71.0)	70 (65.0, 75.0)	70.0 (65.0, 77.0)	<0.001
Male, *n* (%)	160 (47.6)	74 (44.0)	26 (74.3%)	0.005
Smoking, *n* (%)	64 (19.0)	37 (22.0)	14 (40)	0.015
Hypertension, *n* (%)	160 (47.6)	105 (62.5)	21 (60)	0.005
Cystatin C, mg/L	1.01 (0.89, 1.17)	1.05 (0.91, 1.28)	1.14 (1.02, 1.33)	0.002
HCY, μmol/L	12.00 (9.80, 14.80)	13.10 (10.23, 16.75)	14.80 (10.80, 18.90)	0.005
FHS	15.07 ± 0.24	16.55 ± 0.38	17.91 ± 0.63	<0.001

Note. Data presented as mean ± SD or median (Q1, Q3) for continuous variables, and *n* (%) for categorical variables. PWMH, periventricular white matter hypertension; HCY, homocysteine; FHS, Framingham score.

**Table 5:** Univariate analysis of risk factors for the severity of DWMH.

Characteristic	Mild DWMH (*n* = 407)	Moderate DWMH (*n* = 110)	Severe DWMH (*n* = 50)	*p*
Age, year	66.0 (62.0, 71.0)	69.0 (65.0, 74.0)	74.0 (67.0, 82.3)	<0.001
Smoking, *n* (%)	76 (18.7%)	25 (22.7%)	17 (34.0%)	0.036
Hypertension, *n* (%)	197 (48.4%)	63 (57.3%)	34 (68.0%)	0.015
Cystatin C, mg/L	1.02 (0.89, 1.20)	1.05 (0.91, 1.29)	1.15 (1.04, 1.35)	0.001
HCY, μmol/L	12.30 (9.80, 14.90)	12.50 (9.90, 15.85)	15.20 (11.55, 20.05)	0.002
HsTnI, pg/mL	3.50 (2.10, 7.40)	4.50 (2.50, 8.55)	4.80 (3.33, 9.48)	0.001
Proteinuria, *n* (%)	43 (10.6%)	24 (21.8%)	11 (22.0%)	0.002
Uric acid, μmol/L	295.15 ± 4.00	318.13 ± 9.38	313.89 ± 13.50	0.025
FHS	15.10 ± 0.22	16.39 ± 0.38	18.44 ± 0.64	<0.001

Note. Data presented as mean ± SD or median (Q1, Q3) for continuous variables, and *n* (%) for categorical variables. DWMH, deep white matter hypertension; HCY, homocysteine; HsTnI, high-sensitivity troponin I; FHS, Framingham score.

## Data Availability

The data that support the findings of this study are available from the corresponding author upon reasonable request.
